# Online anatomy teaching during the COVID-19 pandemic: assessing the perceptions of undergraduate veterinary medical students

**DOI:** 10.5455/javar.2022.i614

**Published:** 2022-09-30

**Authors:** Reda Mohamed, Lisa A. Benjamin, Pradeep Kumar Sahu

**Affiliations:** 1 Basic Veterinary Sciences, School of Veterinary Medical Sciences, Faculty of Medical Sciences, The University of the West Indies, Eric Williams Medical Sciences Complex, Champ Fleurs, Trinidad and Tobago; 2 Centre for Medical Sciences Education, Faculty of Medical Sciences, The University of the West Indies, Eric Williams Medical Sciences Complex, Champ Fleurs, Trinidad and Tobago

**Keywords:** Anatomy, COVID-19, perception, online classes, veterinary students

## Abstract

**Objective:**

To explore veterinary students’ perceptions of online anatomy teaching during the COVID-19 pandemic.

**Materials and Methods:**

A cross-sectional study was conducted at the School of Veterinary Medicine, where we collected data from year 1 and 2 undergraduate students. A Google Form link to the questionnaire and an introductory message were sent via email to all 68 students who had participated in an online anatomy class between March 2020 and May 2021.

**Results:**

The response rate was 82.2% (56/68). Most students were female (71.43%) and between the ages of 18 and 22 years old (83.93%). Overall, 58.1% of the students found it difficult to learn online anatomy, and most (82%) agreed that it was difficult to learn practical topics online. Nevertheless, 58.9% of the responding students recommended the continued use of online strategies in teaching this subject. Students reported that they could communicate with the lecturer and receive feedback. Perceived benefits of online learning included the convenience of attending class at home and having more available time, as well as the availability of prerecorded videos of course topics.

**Conclusions:**

During the pandemic, students got the opportunity to attend online classes from home. Students thought that online practical anatomy was less useful, and this is an area where new ideas can be suggested to help students see how anatomical structures work.

## Introduction

The anatomy course is an essential component of the medical and veterinary curricula [1–3]. In terms of importance, many programs ranked anatomy first among the other basic subjects [2]. Anatomy is considered one of the most difficult subjects to learn, and certain topics in veterinary anatomy (VA) pose challenges for some students. The primary and most effective method for teaching anatomy is through a combination of didactic lectures, required reading, and cadaveric dissections [4].

The COVID-19 pandemic created unparalleled challenges to anatomy education and forced anatomy educators to rapidly develop alternative strategies to deliver the course content. These strategies included using digital resources and online, synchronous, and asynchronous lectures instead of traditional face-to-face lectures and laboratory sessions, which included osteological specimen banks, prosected specimens, and cadavers [5,6]. In many fields of natural sciences, it is still harder to teach practical sessions online than theoretical parts [7,8].

Mahdy [9] stated that the common problems of students with the online learning of veterinary sciences were the loss of interest, lack of effective communication skills, absence of practical lessons, insufficient availability of online resources, and too little time given to students to do the online tests. Moreover, some recommendations made by students were to improve online learning via providing platforms for online learning, virtual and online resources, videos, and 3D for practical learning, and to increase the time for online tests.

Research reports across the world show that there is a mixed response regarding the effectiveness of online teaching. The present study explored veterinary students’ perceptions of online VA teaching during the COVID-19 pandemic. Further, the study aimed to identify their perceptions of the advantages and disadvantages of the online mode of delivery. The results of this study could help students and teachers figure out how to teach anatomy in the best way possible.

## Materials and Methods

A cross-sectional study was conducted at the School of Veterinary Medicine, The University of the West Indies, St. Augustine, where we collected data from year 1 and 2 undergraduate students. This study was classified as exempt from review (CREC-SA.1011/05/2021) by the Campus Research Ethics Committee of the University of the West Indies. Students were informed about the purpose of the study and were free to withdraw at any time.

### Course taught and assessments

**Figure 1.**
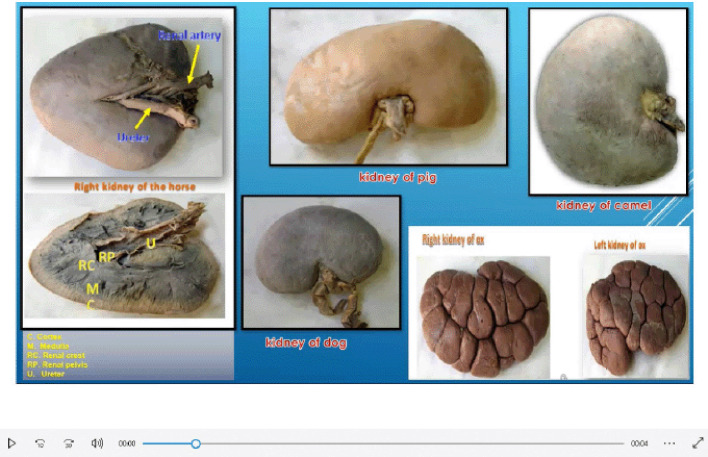
A screenshot of a video prepared as a prerecorded lecture using Screencast-O-Matic software.

**Figure 2.**
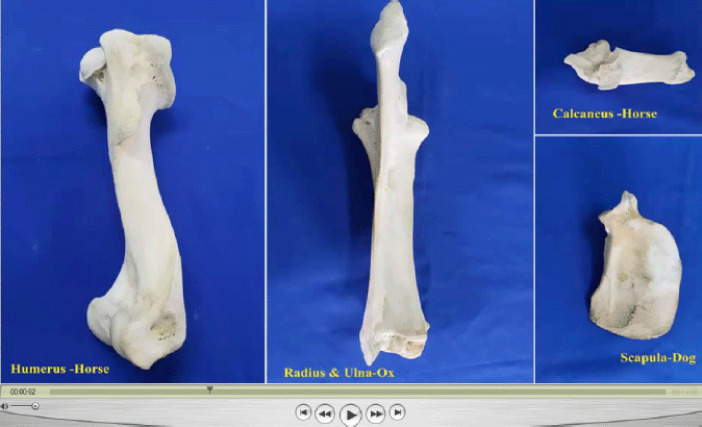
A screenshot of a video prepared as a prerecorded lecture using Screencast-O-Matic software.

Veterinary gross anatomy courses were taught in the first and second years of the (Doctor of Veterinary Medicine) DVM program during the academic year 2020–2021 at The University of the West Indies. The modules of the courses included the limbs, thorax, and abdomen, head and neck, and pelvis of domestic animals, as well as avian anatomy. These courses were delivered online using Blackboard Collaborate (BbC) on the myeLearning platform. The theoretical component of the course was delivered using live streaming and prerecorded lectures (Fig. 1). The practical component of the course was delivered using online dissection videos and live streaming videos (Fig. 2). All necessary safety precautions were taken by the staff to reduce the risk of spreading the SARS-CoV-2. Coursework assessments and final examinations were posted online to students in the form of asynchronous short-answer questions and case scenarios.

### Participants

Surveys were administered to all 68 students over a 1-month period in July and August 2021. Students were of different ages, genders, ethnicities, and nationalities. Each potential survey participant would have participated in an online VA class between March 2020 and May 2021. A test questionnaire was developed and reviewed by four staff members and six students who were not a part of the actual survey. The feedback from these individuals was used to improve the questionnaire.

### Study design and data collection

The administered questionnaire included questions on demographics and student preferences with respect to learning resources and 25 Likert scale questions on students’ experiences in anatomy classes, including the theoretical sessions (lecture) and practical sessions. All the participants could agree or disagree with these items using a 5-point scale. Additionally, three open-ended questions were asked about the challenges, benefits, and suggestions for online anatomy teaching during the COVID-19 pandemic. The demographic information collected included age, gender, and nationality. The questionnaires were administered using Google Forms by forwarding the link to students via email. To maintain the anonymity of the participants in the study, email addresses were not collected from the Google Forms. Data collected was stored on password-protected computers of the investigators for data analysis.

### Statistical analysis

We used Statistical Package for the Social Sciences version 24 software to analyze the collected data. The internal consistency reliability of the questionnaire was measured by Cronbach’s alpha. Descriptive statistics were used to summarize the demographic characteristics of the participants. Percentages were used to determine the distribution of frequencies of responses in each item to show students’ perspectives of online anatomy. The answers to the open-ended questions were put together and grouped by topic to show the pros and cons of learning anatomy online.

## Results

**Figure 3.**
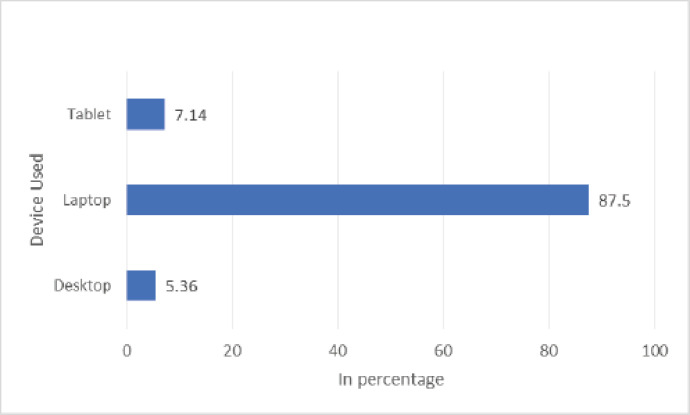
Devices used by the students.

**Figure 4.**
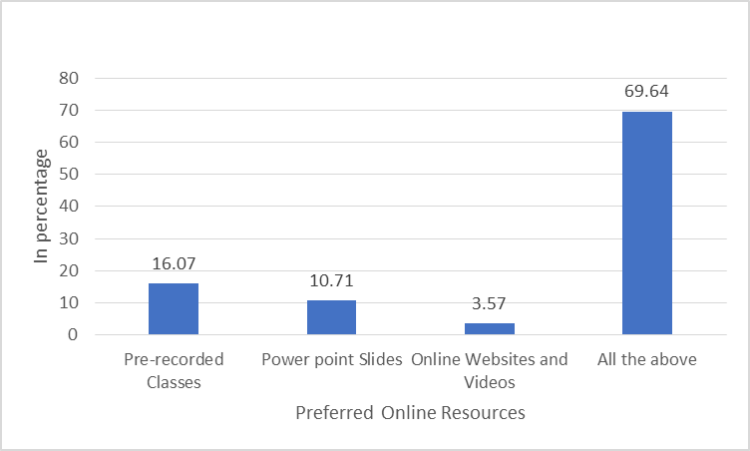
Preferred online resources.

**Figure 5.**
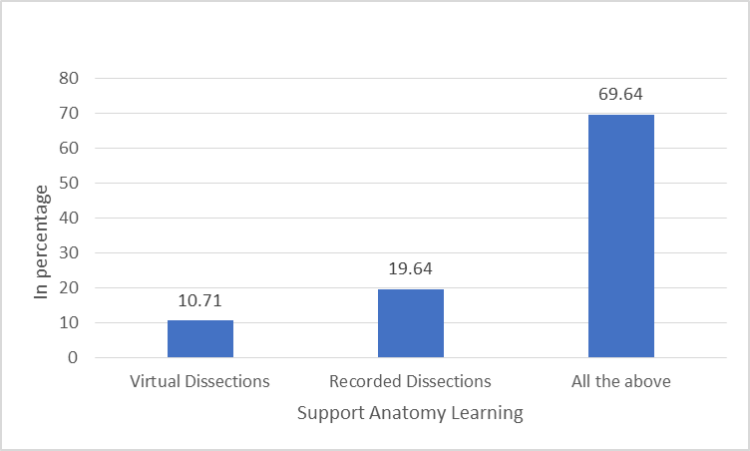
Support for anatomy learning.

The characteristics of the veterinary students are summarized in Table 1. Of 68 students, 56 valid responses were received and used for data analysis, representing a response rate of 82.4%. Among the 56 students, 16 (28.57%) were male and 40 (71.43%) were female. The age of the undergraduate veterinary students ranged from 19 to 30 years (*M* = 21.32), most frequently between 18 and 22 years (83.93%). Of the total, 49 (87.5%) students used laptops during online teaching (Fig. 3). During the session, the preferred learning resources of the students were prerecorded classes, PowerPoint slides given by the teachers, online websites, and videos (Fig. 4). Students preferred virtual sessions. They recorded dissections to support learning in anatomy if COVID-19 becomes endemic (Fig. 5). On average, 24 (42.86%) students spent 1–2 h and 15 (26.89%) students spent more than 2–3 h (per day) outside of scheduled class time studying anatomy during the COVID-19 pandemic. The internal consistency (Cronbach’s alpha) for the questionnaire was found to be 0.92.

**Table 1.** Demographic and academic information of the participants.

Variable	Category	Frequency	Percentage
Gender	Male	16	28.57
	Female	40	71.43
Age	18–20	23	41.07
	21–22	24	42.86
	23 or older	9	16.07
Devices used	Desktop	3	5.36
	Laptop	49	87.5
	Tablet	4	7.14
Preferred online learning resources	Prerecorded classes	9	16.07
	Power point slides	6	10.71
	Online websites and videos	2	3.57
	All the above	39	69.64
Support anatomy learning	Virtual dissections	6	10.71
	Recorded dissections	11	19.64
	All the above	39	69.64
Study hours (outside of classroom)	Below 1 h	7	12.5
	1–2 h	24	42.86
	More than 2–3 h	15	26.89
	Above 3 h	10	17.86

Table 2 reveals the perceptions of veterinary students toward online anatomy teaching during the COVID-19 pandemic. It was found that more than half (57.1%) of the students experienced difficulties while learning anatomy through the online delivery mode. Most of them either agreed or strongly agreed (79.61%) that they had challenges because of a lack of proper devices, low bandwidth, or poor Internet connections. However, most students (71.5%) had good knowledge of technology and were comfortable using these devices during online anatomy teaching. When students were asked about the effectiveness of myeLearning for learning anatomy, they had mixed responses and 44.5% of the students were neutral in their opinion.

Students had a mixed response or were neutral toward a few items. Those were “I found that it was more interesting to learn basic anatomy (theoretical part) online,” “MyeLearning is an effective learning system for anatomy,” “I lacked motivation in the current online anatomy class,” “Online learning of anatomy using prerecorded lectures and virtual models can replace face-to-face teaching,” and “It was easier for me to interact and participate actively during online classes as opposed to face-to-face classes.”

Of the total, most students (67.8%) believed that online demonstrations of dissection and practical videos were comparatively less effective than face-to-face dissection. Furthermore, 82.2% of the students agreed that it was difficult to learn practical topics through online delivery. Students also found it difficult to understand anatomy without handling the specimens (67.9%). More than half of the students did not agree (53.6%) with the statement, “I understand the concepts better when they are taught on an online platform as opposed to face-to-face teaching.”

Concerning curriculum and assessment, there were a few items where the opinion of the students was divided in both negative and positive directions. Those were “I am satisfied with the current online assessment system,” “Regarding the assessment of the practical part of the anatomy course, I would prefer face-to-face exams rather than online exams,” and “The existing anatomy curriculum is suitable for online learning.” Furthermore, regarding assessing the theoretical part of the anatomy course, half of the students preferred online exams. Also, most students (73.2%) found online tutorials (for revisions) useful for learning anatomy.

Students were satisfied with the teachers’ role in online teaching anatomy. Most students (85.5%) agreed that the teachers gave them the necessary feedback in online anatomy classes. More than half of the students (57.1%) agreed that, in online anatomy learning, it was easy for them to communicate information orally in a clear manner. For the item “The teacher clarified my doubts on different topics in anatomy during online learning,” 78.6% of the students expressed their satisfaction. Additionally, the majority of them (80.3%) agreed with the statement “The teacher created a supportive and comfortable learning environment.” In the end, when students were asked if they would recommend the continued use of online strategies in anatomy teaching, 58.9% of the students gave a positive opinion.

### Challenges perceived by students

#### Technical issues

The issue of Internet connection and bandwidth strength was a significant barrier to effective online teaching. Most students reported that they had experienced Internet signal dropping problems for some time during online teaching or assessment. In the initial period when virtual learning was implemented due to the COVID-19 pandemic, there were frequent disruptions in online classroom learning via BbC. It was also difficult to hear some students due to unstable internet connections. One of the students stated, “The challenges I have faced during the anatomy class were either Wi-Fi or laptop issues during classes or exams.”

**Table 2.** Perception of students toward online anatomy teaching during the COVID-19 pandemic.

Item	Strongly disagree (%)	Disagree (%)	Neutral (%)	Agree (%)	Strongly agree (%)	Mean ± SD
I found it easy to adapt to online learning of anatomy.	25	32.1	28.6	3.6	10.7	2.43 ± 1.22
I feel that lack of proper devices, low bandwidth or poor internet connections are barriers to learning.	1.8	8.9	10.7	48.21	31.4	2.05 ± 0.98
I was comfortable with my technological skills during online studying of anatomy (Using computers, surfing the internet, downloading files, etc.).	1.8	5.4	21.4	41.1	30.4	3.93 ± 0.95
I found that it was more interesting to learn basic anatomy (theoretical part) online.	8.9	17.9	28.6	32.1	12.5	3.21 ± 1.16
MyElearning is an effective learning system for anatomy	3.6	10.7	44.5	28.6	12.5	3.36 ± 0.96
The materials provided during online teaching were enough for learning anatomy.	0	14.3	33.9	39.3	12.5	3.50 ± 0.89
Online demonstration of dissection and practical videos are comparatively less effective than face-to-face dissection.	3.6	3.6	16.1	48.2	28.6	2.05 ± 0.96
It is difficult to learn practical topics through online delivery.	3.6	5.4	8.9	42.9	39.3	1.91 ± 1.01
I find it difficult to understand anatomy without handling the specimens.	7.1	1.8	23.2	30.4	37.5	2.10 ± 1.15
I lacked motivation in the current online anatomy class.	10.7	17.9	12.5	32.1	8.9	2.54 ± 1.35
Online learning of anatomy using pre-recorded lectures and virtual models can replace face-to-face teaching.	16.1	30.4	26.8	17.9	8.9	2.73 ± 1.20
I understand the concepts better when they are taught on an online platform as opposed to face-to-face teaching.	25	28.6	32.1	**7.1**	**7.1**	2.42 ± 1.16
It was easier for me to interact and participate actively during online class as opposed to face-to-face classes.	14.3	26.8	26.8	23.2	12.5	2.95 ± 1.26
I am satisfied with the current online assessment system.	8.9	17.9	42.9	17.9	12.5	3.07 ± 1.11
With reference to assessment of the theoretical part of the anatomy course, i would prefer face-to-face exams rather than the online exams.	23.2	26.8	35.7	8.9	5.4	3.54 ± 1.11
With reference the assessment of the practical part of the anatomy course, i would prefer face-to-face exams rather than online exams.	16.1	16.1	26.8	32.1	8.9	2.98 ± 1.23
The existing anatomy curriculum is suitable for online learning.	5.4	17.9	46.4	21.4	8.9	3.11 ± 0.98
I found online tutorials (for revisions) are useful for learning anatomy.	0	3.6	23.2	41.1	32.1	4.02 ± 0.84
The teacher provided necessary feedback in online anatomy class.	0	1.8	12.5	42.9	42.9	4.27 ± 0.75
In online anatomy learning, it was easy for me to communicate information orally in a clear manner.	3.6	12.5	26.8	37.5	19.6	3.57 ± 1.06
The teacher clarified my doubts on different topics on anatomy during online learning.	0	1.8	19.6	39.3	39.3	4.16 ± 0.80
The teacher created a supportive and comfortable learning environment.	1.8	0	17.9	48.2	32.1	4.09 ± 0.82
I would recommend continued use of online strategies in the teaching of anatomy.	0.4	12.5	23.2	39.3	19.6	3.55 ± 1.11

#### Lack of motivation

It was stated by some of the students that they were less motivated toward online anatomy learning because of the prolonged screen time. Students felt it was hard to pay attention and focus during online learning because of the large volume of information covered in the anatomy course. They also did not get the opportunity to see and interact with their classmates physically. Therefore, they did not put as much effort into participating in the course as they would have done if it was a regular face-to-face class and they were physically in a classroom. One of the students believed: “The environment hinders our learning instead of being in a controlled environment such as a classroom, which puts a student automatically into the mood, mindset, and restores our motivation to learn.”

#### Difficulties coping with online labs

Students did not have any problems with lectures via online delivery mode. However, it was found that most students had expressed their dissatisfaction regarding online labs. Students believed that online practical sessions were not helpful; it is hard to learn on a laptop, especially when there is a hefty practical aspect to the subject. They said that it was difficult to understand the orientation of certain slides without seeing a specimen in a face-to-face class. One of the students said, “The online labs were a little hard because I couldn’t hold the specimen in my hands and figure out what it was by myself.”

### Benefits of online anatomy teaching

#### Learning from home

During the COVID-19 pandemic, when face-to-face learning was not possible, students got the opportunity to learn anatomy and other subjects from their homes via online mode. Since they did not have to travel to class, it was easier for them to log in and attend class on time. Students felt that it was easy and less stressful to get to online classes, and they found it more comfortable and relaxing. One of the students stated that, “It was easier to pay attention while being in the comfort of one’s own home, and thus I was personally able to learn and grasp concepts better without external distractions.”

#### Asynchronous learning

Students expressed their satisfaction with the prerecorded videos. They also had access to the recordings of the lectures in the BbC. The prerecorded videos were great in that they helped students be better prepared for the actual live session. Students could always refer to prerecorded videos if an important point was missed during class. Prerecorded videos were useful for the students during the revision of the topics. As one student said, “Prerecorded lectures gave me opportunities to make notes beforehand and ask questions about any uncertainties during class and get clarification.”

#### Handling of devices

Students were quick learners in terms of handling computers, laptops, and other devices used for online learning. With some initial challenges, they learned to use BBC through the university’s myeLearning platform. All the students knew how and when to mute and unmute the microphone, chat during discussion, download and upload materials, and share screens. Due to the proper handling of devices, there were fewer distractions during the teaching. Additionally, students also appreciated the teachers for their efforts in managing both the theoretical and practical parts of the anatomy course. There was clear communication between the lecturer and the student. Students felt more comfortable clarifying their doubts in virtual mode. It was quite easier to voice opinions and concerns during class time.

## Discussion

In the 2020–2021 academic year, most of the VA courses at the SVM were online. This report is an evaluation of the perceptions of students of veterinary medicine about these online VA classes that were conducted during the pandemic. Like veterinary students in Portugal [10], the respondents thought that not having to travel to class saved them time and made it easier for them to be on time and less stressed.

### Intention to use

Overall, 58.9% reported that they would recommend the continued use of online strategies to teach VA. However, there was a neutral response to the question, “Can online learning using prerecorded lectures and virtual models replace face-to-face teaching?” During the pandemic, the students at Liaquat College of Medicine and Dentistry did not like it when e-learning took the place of face-to-face learning [11].

It is conceivable that the perceived usefulness of online learning for students can be diminished by a lack of access to computers [12,13]. All students in the current study had access to at least one device, most frequently a laptop. No one said they used a smartphone to get into the course, unlike students in South Africa and other places [9,14].

It is important for online learning to have a stable Internet connection with sufficient bandwidth to allow for the streaming of meetings, webinars, videos, or the download of images and text. Most students reported that they had experienced an Internet signal-dropping problem during online teaching or assessment. Internet-related difficulties were also reported by other students in the Caribbean and elsewhere [9]. Like students in other countries, survey participants felt that lack of proper devices, low bandwidth, or poor Internet connections acted as barriers to learning [15–17]. Identified barriers should be addressed with urgency. Advice should be provided to help students select appropriate devices.

### Perceived ease of use

The influence of computer self-efficacy on the perceived ease of use of online learning and the intention to use the technology is worthy of further investigation in the developing country context [18]. In the current study, although the majority of students (71.5%) believed that they had good knowledge of technology, many found it difficult to adapt to online learning. The finding that a high percentage of veterinary students were comfortable with their technology skills was similar to that reported for 82% of veterinary students from 87 countries and 6 continents who studied online when restrictions on onsite classes were in place [19]. Students can be made to feel comfortable with the use of technology in a class by providing tutorials or tips at the start of the semester [20].

### Perceived usefulness

Although the VA course is conducted during the first 2 years of the DVM at the institution where the current study was conducted, the knowledge and skills gained in this course are applied later in the paraclinical and clinical years when students take subjects such as Surgery, Pathology, and Meat Inspection. A recent report on how veterinary surgeons use anatomy in their work confirmed that anatomy is used every day in different parts of practice. This is because veterinary surgeons must have a good understanding of the body’s structures and how they fit together in space [1].

Perceptions about how easy it is to use online learning differed between the theoretical and practical components of VA. In the current study, most students found it difficult to learn practical topics online and to understand VA without handling specimens. This is consistent with previous reports in which students expressed difficulty with practical online anatomy as it was challenging to appreciate the spatial relationship of structures in practical VA [4,10,20]. The 3D printed scan is one of several tools which can be used to facilitate learning in this area [4]. Traditionally, dissection has been a major component of the practical aspect of anatomy. However, the approach to the use of cadaveric dissection, kinesthetic learning, has been ambivalent in recent times, with some medical schools discontinuing and then reintroducing dissection in recent years, preceding the COVID-19 pandemic [21], highlighting the interplay between the challenges experienced with this practice, such as protests by animal welfare organizations, and the perceived benefits of face-to-face dissection, such as tactile learning and teamwork.

The dichotomy in attitudes toward theoretical and practical VA extended to the assessments. Most students were happy with the current online assessment system for theoretical VA, but they were split on whether they would rather take practical VA exams in person or online.

### Success factors (motivation, participation, class attendance, and feedback)

Online learning is inherently student-centered. In addition to self-efficacy, success in online learning requires self-regulation and self-direction. The students participate in problem-based learning sessions from year 1, in which they are regularly assessed for behavior related to self-regulation and self-direction. According to Araka et al. [22], tailored feedback, instructor guidance, no student–instructor or peer interaction, and no automation tools were challenges to improving self-regulation in students in the online learning environment in Kenya. In the current study, questions about feedback from lecturers in the course received positive responses from students.

The asynchronous resources provided to students allowed them to review the concepts as many times as they needed to understand. The learning resources preferred by the students in the current online teaching were prerecorded classes, PowerPoint slides given by teachers, online websites and videos, and virtual and recorded dissections to support learning in anatomy.

### Limitations

Although the response rate of this survey was high, at 82.4% (56/68), the class size was small. In addition, the students were from one campus of a university that has only one school of veterinary medicine. In the future, a survey can be conducted across regional schools of veterinary medicine that share some of the challenges experienced by students in identifying resources and possible synergies related to online learning.

A limitation of this study is that students’ perceptions were not compared to student examination scores. Yoo et al. [23] found that students who took anatomy tests in 2020 after taking online lectures and blended dissection labs did better than students who took anatomy tests in 2019 after taking traditional lectures and labs (cadaver dissection). [23] Examinations were face-to-face in both 2019 and 2020. The cohorts of students who received online delivery of lectures should be followed, and their experiences should be assessed in the clinical years when they apply the knowledge gained in the VA course to determine if intervention is needed as far as their prerequisite knowledge of anatomy is concerned.

## Conclusions

Students were divided in their responses to the statement “The existing anatomy curriculum is suitable for online learning,” with the highest group being neutral (46.4%). This survey can also provide information that can be used to develop recommendations for the Curriculum Committee to increase the agility of the curriculum by providing a route for a successful response to be mounted if there is a need to switch to fully online delivery of the course at short notice at any time in the future. In the future, it might be possible to use virtual environments, 3D images, artificial intelligence, and robots to practice performing technical procedures on different kinds of animals.

## List of Abbreviations

BbC: Blackboard Collaborate; DVM: Doctor of Veterinary Medicine; VA: Veterinary anatomy.
